# Prevalence of mobility limitations among the elderly in Saudi Arabia: A secondary analysis of the 2025 Elderly Survey

**DOI:** 10.1097/MD.0000000000051152

**Published:** 2026-10-16

**Authors:** Saad Z. Alqahtani

**Affiliations:** a Department of Physical Therapy, College of Medical Rehabilitation Sciences, Taibah University, Madinah, Saudi Arabia.

**Keywords:** elderly, mobility limitations, Saudi Arabia, stair climbing, walking

## Abstract

Mobility limitations in terms of walking and stair climbing among the elderly are associated with limitations in activities of daily living (ADL), higher annual healthcare expenditure, more hospitalizations, and increased mortality. However, evidence on the prevalence of mobility limitation among the elderly in Saudi Arabia remains limited. Therefore, this study aimed to investigate this issue using data from a national public survey. Data were obtained from the 2025 Elderly Survey, which was designed and administered by the General Authority for Statistics (GASTAT) of Saudi Arabia. Secondary analysis was applied. The survey included both Saudi and non-Saudi residents from 13 administrative regions across the kingdom (n = 32,745 households, which yielded a weighted national population of approximately 1,758,737 individuals). In total, 42% of the individuals were aged 60 to 64 years, and 25.5% of the individuals experienced mild difficulty in terms of mobility, specifically walking and climbing stairs, whereas 10.8% experienced severe difficulty. Furthermore, 61.8% of the individuals were not physically active. Additionally, 44.7% of the individuals had multiple chronic conditions, 25.5% had 1 chronic condition, and 29.8% had no chronic conditions. The survey data indicated that more than one-third of the elderly in Saudi Arabia have mild-to-severe mobility limitations in terms of walking and climbing stairs.

## 1. Introduction

Mobility limitations, manifested as difficulty in walking a quarter mile or even several steps, is often present in the elderly population. The prevalence of mobility limitations in individuals aged≥65 years is well documented in literature; percentages range from 8.5% to 28% among different populations.^[1,2]^ Disability often begins with an inability to perform physical tasks or mobility difficulties, and elderly individuals with physical limitations are at a higher risk of developing disability, which manifests as dependency with respect to activities of daily living (ADL), increasing the risks of hospitalization and mortality.^[3,4]^ Mobility limitations specifically related to walking and stair climbing among the elderly are associated with increased mortality, limitations in ADL, higher annual healthcare costs, and more hospitalizations.^[2,5]^ Furthermore, the severity of mobility difficulties is associated with the probability of fall recurrence.^[6]^ Thus, difficulties in mobility increase the risk of frailty and adversely affect health outcomes later in life.^[6]^ Several factors have been associated with mobility difficulties, such as age >60 years, low cognitive scores, presence of multiple comorbidities, and hearing loss.^[6]^ Sarcopenia is also associated with mobility limitations in the elderly.^[7]^ Low protein intake is associated with mobility-related limitations, which may reflect disrupted interactions between the neural and muscular pathways.^[8]^

From a clinical perspective, once a disability develops, it is difficult to inhibit functional decline; hence, preventing further worsening is a priority for rehabilitation.^[4]^ Therefore, early identification of individuals at higher risk is necessary to implement preventive measures.^[4]^ Mobility limitations in the lower extremities increase considerably after the age of 60 years.^[6]^ In 2021, the World Health Organization estimated the life expectancy in Saudi Arabia to be more than 70 years, indicating an increasing trend in lifespan over the past 2 decades.^[9]^ A recent study reported on the prevalence of mobility in Saudi Arabia among people aged ≥15 years.^[10]^ However, evidence regarding mobility limitations in the elderly population of Saudi Arabia remains limited. Hence, this study aimed to estimate the prevalence of mobility limitations among the elderly in Saudi Arabia based on data from a national public survey.

## 2. Methods

The study data were obtained from the 2025 Elderly Survey of the General Authority for Statistics (GASTAT). This is a publicly available statistical resource produced by a Saudi Arabian government authority responsible for reporting economic, social, and environmental statistics at the national level on a periodic basis. The data were obtained from the 2025 survey of the elderly. The data were collected from January 26 to March 1, 2025. The data were publicly available and aggregated. Moreover, GASTAT confirmed data anonymity using 2 measures: removal of direct information such as names, addresses, contact numbers, and identification numbers; and combining different rare characteristics of respondents (e.g., age, occupation, education) to prevent identification. The 2025 Elderly Survey was conducted by GASTAT, Saudi Arabia’s official statistical authority, in accordance with the laws and regulations governing its statistical activities and authorizing GASTAT to collect statistical data and conduct surveys. When beginning the questionnaire, the respondents were informed about the purpose and official basis of the survey, and that their information would remain confidential and be used for statistical purposes only. Therefore, no separate written informed consent form was provided as part of the survey questionnaire. In addition, the secondary analysis performed in this study received institutional ethics approval from the Institutional Review Board of the College of Medical Rehabilitation Sciences at Taibah University with an Ethical Approval number (TU-RHB-26-09-002).

### 2.1. Questionnaire test

The survey was designed and prepared by specialists from GASTAT. During survey preparation, a cognitive test was conducted to assess whether the questions were understood as intended and clearly worded, respondents could answer them effectively, the response options were appropriate, and respondents were willing to answer openly. Interviews were also conducted with a random sample of households across the country, with education level, sex, and nationality taken into consideration. The questionnaire comprised 4 sections: demographic data, health status and difficulties, social activities and communication, and quality of life and general satisfaction. The data were collected through computer-assisted personal interviews (CAPI). Trained staff using electronic devices asked the questions using the online survey; they entered the answers into the relevant forms simultaneously. The interviewers were trained in accordance with GASTAT’s standard training procedures. The training was conducted over 3 days and included both theoretical and practical sessions covering the questionnaire and its concepts, interviewing techniques, data review and validation, and the use of the CAPI data-collection system. The training concluded with an assessment to evaluate the interviewer’s understanding and readiness for fieldwork. Interviewers who met the GASTAT requirements and passed the assessment were selected for fieldwork. The 2025 Elderly Survey questionnaire was available in both Arabic and English. Interviews were primarily conducted in Arabic, with English used when preferred by the respondent. Both language versions followed the same questionnaire structure, response categories, and validation rules.

### 2.2. Definitions

GASTAT used the following definitions. The elderly were defined as “individuals who had reached the age of ≥60 years, according to the definition of the Law on the Protection of the Elderly issued by the Council of Ministers”; a household was “an individual or group of individuals who are or are not bound together by ties of kinship, and who share residence and food in 1 dwelling during the time of survey.” Difficulty was defined as “a deficiency or lack in a person’s ability to perform any activity within normal limits, such as a deficiency in hearing”; Physical Activity was defined as “any physical effort performed by an individual to move the body and contribute to improving general health, such as walking or light exercise.” Mobility limitation was referred to difficulty in walking and climbing stairs. The mobility item followed the Washington Group methodology^[11]^ for measuring functional difficulty. Walking and climbing stairs were assessed as a single item. The question was “Do you experience difficulty walking or climbing stairs?” with the following response categories: 1 – no difficulty, 2 – some difficulty, 3 – a lot of difficulty, 4 – cannot do at all. The classification of functional difficulty and disability was based on the Washington Group methodology, and the abovementioned 4 response categories were used in the questionnaire.

The responses were then classified into 3 categories as follows:

No difficulty: Respondents who selected “No difficulty”
Mild difficulty: Respondents who selected “Some difficulty.” This was deemed to indicate the presence of a functional difficulty that does not meet the threshold used to classify disability.
Severe or extreme difficulty (disability): Respondents who selected “A lot of difficulty” or “Cannot do at all.”

### 2.3. Sample design

Eligibility was defined as any individual aged ≥55 years who was residing in the sampled household, regardless of nationality or sex. No additional exclusion criteria were applied beyond the age and household residency requirements. All household members who met the eligibility criteria were selected to complete the Elderly Survey questionnaire. If a household had 1 or more eligible individuals aged ≥55 years, each eligible individual was selected, and the questionnaire was completed for each of them. No additional within-household selection procedure was applied. Proxy responses were permitted under specific conditions when an eligible individual was unable to respond personally because of illness, disability, or difficulty understanding or responding to the survey questions.

In such cases, the designated household respondent, according to the GASTAT household survey procedures, provided the information on behalf of the eligible individual. The enumerators were trained on how to administer the questions and handle proxy-response cases. The questionnaire also included validation and consistency checks to identify errors or inconsistent responses during data collection.

The 2025 Elderly Survey sample was designed in 2 phases. Initially, a random sample of the primary enumeration areas was selected, followed by the selection of a systematic random sample of households within each chosen initial enumeration area.

To increase sampling efficiency, national representation, and accurate results, the enumeration areas were used as primary sampling units that were subsequently divided into homogeneous strata based on governorates as actual strata, where the number of governorates in the kingdom is 150. Subsequently, the total sample size was estimated at the country’s level, using proportional to size allocation to all administrative regions. Additionally, adjustments were made to ensure that the sample size of the small governorates did not fall below the specified minimum. Furthermore, several sample size parameters and determinants were used to ensure an appropriate sample size across the country: the coefficient of variation (CV) was <1.8%, the expected response rate was 65%, and the proportion estimate was based on a 95% confidence level (1–α = 0.95). These parameters yielded a sample of 32,745 households from 150 governorates across 13 administrative regions of the country (Table 1).

**Table 1:** Distribution of the sample at the level of administrative regions.

ID	Administrative region	Number of enumeration areas	Number of households
1	Riyadh	295	4425
2	Makkah	309	4635
3	Madinah	230	3450
4	Qassim	140	2100
5	Eastern Region	304	4560
6	Aseer	173	2595
7	Tabuk	136	2040
8	Hail	102	1530
9	Northern Borders	75	1125
10	Jazan	138	2070
11	Najran	103	1545
12	Al-Baha	91	1365
13	Al-Jouf	87	1305
Total		2183	32,745

### 2.4. Statistical processing

Several stages of processing were carried out to ensure data accuracy and quality. These included field verification during data collection using the CAPI system, which incorporates real-time validation to avoid the entry of missing and illogical data. After data collection, a desk review was performed using a logical review to verify response consistency (e.g., alignment of the ability to perform activities and individuals’ health status), along with range of data values and missing data. Statistical checks were performed by examining data distribution, means, and deviations to detect abnormal patterns as well as illogical values. Finally, the data were also compared to data from the kingdom population census carried out by the GASTAT to confirm spatial and temporal consistency. The survey sample included 32,745 households; within these households, 23,164 eligible elderly individuals had valid completed responses. This represents the actual unweighted number of elderly respondents before applying survey weights. All completed questionnaires underwent validation and quality checks before the production of the published indicators.

### 2.5. Missing values

A limited number of missing values were identified in some variables and were imputed using an Ordinal Logistic Regression model, based on the ordinal nature of the affected variables. Responses outside the predefined valid response range were reviewed separately. Original values were recovered where possible, while the remaining cases requiring statistical treatment were imputed using a Bayesian model. The final analytical dataset included valid completed responses after the validation and imputation process. Unweighted denominators were generally consistent across indicators, with differences mainly due to question applicability and skip patterns.

### 2.6. Imputation and calibration

The design weight was used to calculate sample weights of the selected unit (i.e., individual) based on the following equation:


$$\begin{array}{c} w_{hi}^{I}\;=\;\frac{1}{\pi_{hi}^{I}}i \end{array}$$

Where $\pi_{hi}^{I}$ 𝒊 represents the probability of selecting the individual from stratum *h*.

Non-response weight adjustment:

An adjustment after data collection was made to compensate for non-response or missing data, which was calculated using the following equation:


$$\begin{array}{c} w_{h}^{*}\;=\;w_{h}\;\times \;f_{hNR} \end{array}$$

Where $w_{h}^{*}$ represents the adjusted weight for non-response in stratum (or adjustment category) h, and the correction factor for each stratum (or adjustment category) is calculated $f_{hNR}$ as follows:


$$\begin{array}{c} f_{hNR}\;=\;\frac{\sum_{hi\in R}w_{hi}\;+\;\sum_{hi\in NR}w_{hi}}{\sum_{hi\in R}w_{hi}} \end{array}$$

Where NR represents the non-responses and R represents the responses. The final outcomes are presented in Table 2.

**Table 2:** Survey response outcomes after the data collection process completed.

Administrative region	Provided all data	Nonresponsive	Total
Riyadh	4322	72	4425
Makkah	4246	107	4635
Madinah	2775	554	3450
Qassim	2013	80	2100
Eastern Region	4199	314	4560
Aseer	2208	333	2595
Tabuk	1960	38	2040
Hail	1308	169	1530
Northern Borders	1042	69	1125
Jazan	2018	44	2070
Najran	1542	3	1545
Al-Baha	1327	32	1365
Al-Jouf	1205	89	1305
Total	30,165	1904	32,745

Weight calibration:

Sample weight adjustment was made to confirm that the collected data were aligned with overall population specific characteristics based on the following equation:


$$\begin{array}{c} W_{f}\;=\;W_{1}\;*\;\frac{x}{\sum W_{1}} \end{array}$$

$W_{f}$: Final weight.

x: Total number of individuals in the calibration category by population projection.

$\sum W_{1}$: Total adjusted weights for individuals in the calibration category. Therefore, the previous calculations yielded a weighted national population of approximately 1,758,737 individuals. Therefore, the number of individuals (i.e., 1,758,737) in this study is the weighted national population representing the elderly in Saudi Arabia.

The aforementioned methods used for data collection and statistical processing, including error detection, imputation, and calibration, and the overall accuracy of the data, are published on the GASTAT website.^[12]^ The publicly available data were aggregated; therefore, inferential statistical analysis could not be performed. Data are presented descriptively in tables as percentages and total numbers as individual counts from the public published results from the GASTAT website. These numbers and percentages were taken from the GASTAT website without any modifications. These data sets were accessed on August 6, 2026. The combined percentages of mild and severe difficulty (Fig. 1) were calculated manually. In addition, percentages were extracted from tables where only individual numbers that were provided. In this process, we converted the number of individuals of some variables to percentages based on total individuals of the same table (Tables 1 and 2).

**Figure 1.**
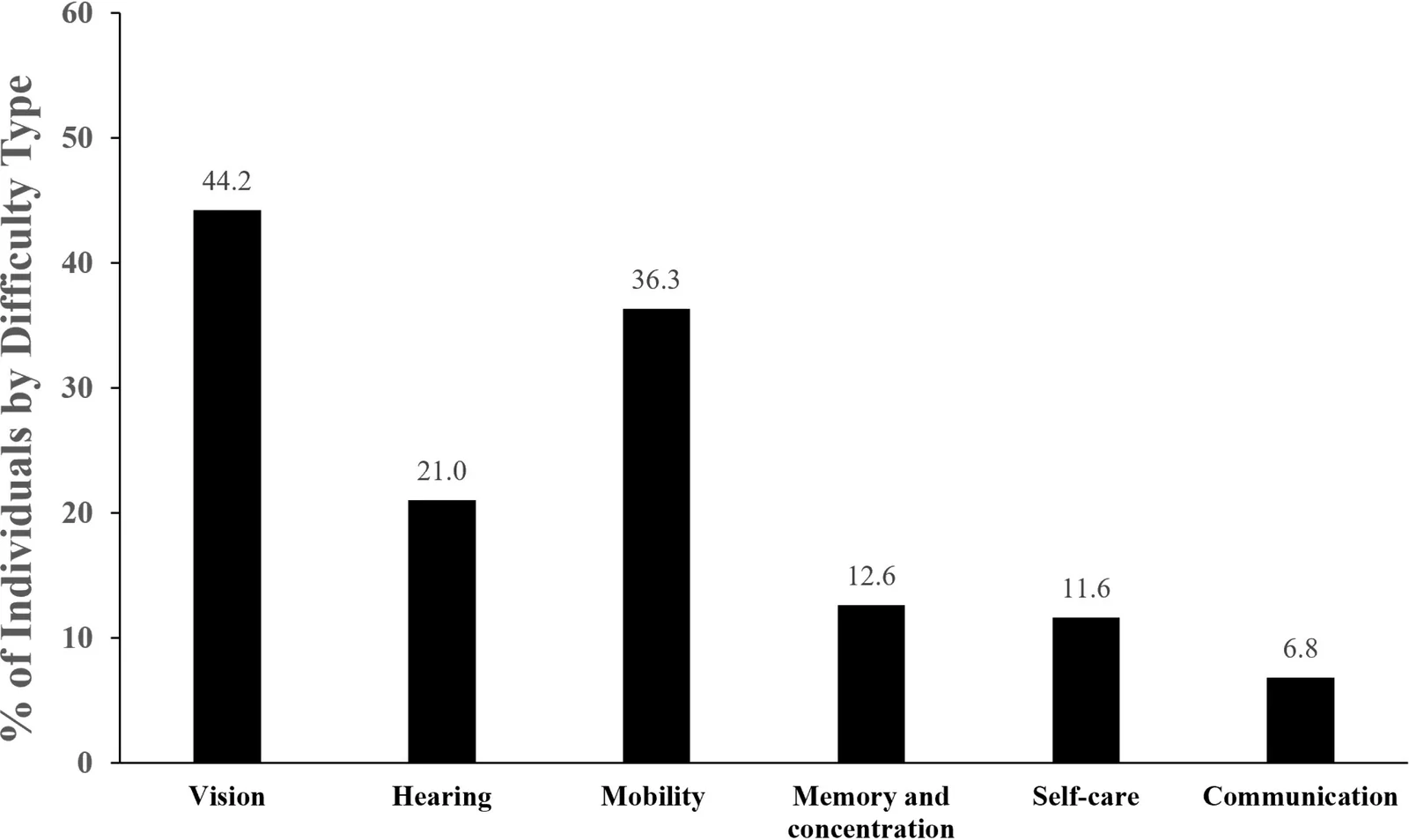
Percentage of individuals aged ≥60 years stratified according to type of difficulty. Note: Bars represent the combined percentages for mild and severe difficulty.

## 3. Results

The results are based on an analysis of data from the 2025 Elderly Survey of 1,758,737 individuals based on the weighted national population, both Saudi and non-Saudi residents, from 13 administrative regions across the kingdom (Supplementary File, Supplemental Digital Content 1). Overall, 1,007,238 individuals were men and 751,499 were women, with a male-to-female ratio of 1.3. Approximately 42% of the individuals were aged 60 to 64 years. Furthermore, approximately 75% of the individuals were married, and 30.1% had no formal education (Table 3).

**Table 3:** Demographic data of the participants.

	Male	Female	Total
Age group, yr			
60–64 (n)	4,46,919	2,87,630	7,34,549
65–69 (n)	2,65,020	1,93,297	4,58,317
70–74 (n)	1,33,568	1,12,704	2,46,272
75–79 (n)	78,923	70,233	1,49,156
80–84 (n)	43,218	44,669	87,887
≥85 (n)	39,590	42,966	82,556
Marital status			
Married (n)	8,90,485	4,34,855	13,25,340
Divorced (n)	47,985	48,601	96,586
Widowed (n)	58,347	2,64,206	3,22,553
Never married (n)	10,421	3837	14,258
Education level			
No formal education (%)	18.1	46.1	30.1
Primary education (%)	18.9	20.8	19.6
Intermediate education (%)	14.7	10.7	13.0
Secondary education (%)	20.4	11.2	16.4
Associate degree (%)	4.9	1.7	3.6
Higher education (%)	23.0	9.5	17.3

Table 4 summarizes the presence of chronic conditions. The percentage of individuals with multiple chronic conditions was approximately 38.2% for men and 53.6% for women; 35.2% and 22.4% of men and women did not have chronic conditions, respectively. The presence of only 1 chronic disease was seen in 26.6% and 24% of men and women, respectively.

**Table 4:** Percentage distribution of chronic disease status and type, and self-rated health status.

	Male	Female	Total
Chronic disease status			
No chronic conditions	35.2	22.4	29.8
One chronic condition	26.6	24.0	25.5
Multiple chronic conditions	38.2	53.6	44.7
Chronic disease type			
Arthritis	14.4	31.4	21.7
Asthma/ respiratory allergy	5.0	8.2	6.4
Cancer	0.9	1.6	1.2
Dementia/ Alzheimer disease	1.2	1.7	1.4
Diabetes	42.6	47.8	44.8
Chronic kidney disease	2.4	1.8	2.1
Heart disease	8.9	8.5	8.7
Chronic lung disease	1.2	2.8	1.9
Stroke	2.1	1.7	1.9
Obesity	2.7	5.2	3.8
Hypertension	39.5	50.8	44.3
Neurological disorders/mental illness	1.0	1.8	1.4
Other long-term health conditions	1.5	2.9	2.1
No diagnosed disease	35.2	22.4	29.8
Self-rated health status			
Excellent	24.9	13.7	20.1
Very good	22.0	16.6	19.7
Fair/ moderate	45.1	57.1	50.2
Poor	6.8	10.8	8.5
Very poor	1.2	1.8	1.5

The disease distribution was as follows: diabetes: 42.6% and 47.8% of men and women, respectively; hypertension: 39.5% and 50.8% of men and women, respectively; arthritis: 14.4% and 31.4% of men and women, respectively. Lastly, no diagnosed disease was found in 35.2% and 22.4% of men and women, respectively.

Regarding the mode of transportation used, 39.8%, 31.2%, and 15.9% of respondents, respectively, reported that they usually traveled with others, drove their own car, and walked (Table 5).

**Table 5:** Percentage distribution of usual means of transportation.

Mode of transportation	Male	Female	Total
Walking	18.9	11.4	15.9
Driving own car	49.9	4.1	31.2
Private driver	6.4	13.8	9.4
Traveling with others	21.8	65.8	39.8
Ride-hailing services (apps)	3.0	4.9	3.7

Regarding mobility, 25.5% of the individuals had mild difficulty in walking and climbing stairs, whereas 10.8% had severe difficulty (Table 6). However, when the percentages of individuals experiencing mild and severe difficulty were combined for each difficulty, nearly half of the elderly had vision difficulties (44.2%), and more than one-third of the elderly had mobility difficulties (36.3%, Fig. 1).

**Table 6:** Percentage distribution by type and severity of difficulty/disability.

Difficulty	No difficulty (%)	Mild difficulty (%)	Severe or extreme difficulty (disability) (%)
Vision	55.8	38.1	6.1
Hearing	79.0	17.4	3.6
Mobility (walking and climbing stairs)	63.7	25.5	10.8
Memory and concentration	87.4	10.1	2.5
Self-care	88.4	8.1	3.5
Communication	93.2	5.2	1.6

The total percentage of individuals aged ≥60 years who were not physically active was 61.8%. In addition, the percentage was higher in women than in men (74.9% vs 51.9%). Among the listed activities, walking or running was the most common (37.6%; Table 7).

**Table 7:** Percentage of individuals who practiced physical activities regularly by sex and type of physical activity.

Physical activity	Male	Female	Total
Walking/running	47.3	24.7	37.6
Swimming	2.3	0.6	1.6
Other activity	2.9	0.4	1.8
Not physically active	51.9	74.9	61.8

## 4. Discussion

This study explored the mobility limitations experienced by the elderly in Saudi Arabia based on data from a national survey. The study revealed that more than one-third of the elderly in Saudi Arabia had mild or severe mobility difficulties (36.3%). This finding is in line with a previous study reported that 31.7% of adults aged ≥65 years had difficulty walking 3 blocks, and 30.2% had difficulty climbing 1 flight of stairs.^[13]^ The results of the previous study were presented from the longitudinal Survey of Income and Program Participation (i.e., wave 5 2025) of the United States. The methods were similar to our study, such as using the CAPI system. Moreover, the responses were weighted to the actual population at the interview time, sampling weights were applied to create national estimates, and self-reporting questions were used to examine mobility limitations (i.e., walking 3 city blocks and climbing a flight of stairs). However, the mobility limitation questions used in our study only asked about the level of difficulty in walking and climbing stairs in general.

Another study reported that the percentage of elderly people aged ≥65 years using the self-reporting question (“How much difficulty do you have walking a quarter of a mile – that is, about 2 or 3 blocks?”) were weighted 28%; 17% had difficulty in and inability to walk a quarter of a mile, respectively.^[2]^ Similarly, in our study, weighted 25.5% and 10.8% had mild and severe difficulty, respectively. The previous study was similar to our study in several aspects. One was the use of self-reporting questions. Second, data from a Medicare Current Beneficiary Survey, which is a nationally representative survey, were used. Furthermore, sampling weights of the data were used in the analyses. Moreover, individual responses of “a little,’ “some,” and “a lot” of difficulty were collapsed into 1 category “any difficulty,” and inability to do it; this resulted in only 2 categories, while in our study we have mild and severe difficulty.

This study provides national descriptive evidence on mobility limitations among the elderly in Saudi Arabia. Some implications and recommendations can be drawn from these findings. As mobility limitations increase in both sexes with advanced age,^[14]^ longitudinal studies using objective measures in older populations are needed to explore the prevalence and risk factors of mobility limitations among the elderly in Saudi Arabia. However, given that the risk factors of mobility limitations are multidimensional, including environmental, organizational, and social components,^[15]^ future studies using multidimensional designs are warranted. In addition, interventions to prevent mobility limitations among the elderly should prioritize lower-extremity function to preserve critical functions, such as walking^[6]^; therefore, clinical settings should prioritize such preventive interventions.

This study has some limitations. The data were self-reported and lacked objective measurements, which may have introduced reporting bias. However, from a clinical perspective, a self-reported mobility survey is a simple and cost-free tool that healthcare providers can use to identify significant risk factors and conditions.^[2]^ In addition, the cross-sectional design limits the ability to draw causal relationships. Furthermore, the use of aggregated data precluded inferential statistical analysis and possible selection and reporting bias, as well as uncertainty regarding non-response, limiting the ability to assess determinants of mobility limitation.

## 5. Conclusions

The data obtained in the current study indicated that more than one-third of the elderly in Saudi Arabia had mild-to-severe mobility limitations in terms of walking and climbing stairs. However, the data were based on aggregated and self-reported answers; therefore, the results should be interpreted with caution.

## Acknowledgments

I would like to thank Editage (www.editage.com) for English language editing.

## Author contributions

**Conceptualization:** Saad Z. Alqahtani.

**Formal analysis:** Saad Z. Alqahtani.

**Writing – original draft:** Saad Z. Alqahtani.

**Writing – review & editing:** Saad Z. Alqahtani.


