# Supplementary material for: Prevalence of mobility limitations among the elderly in Saudi Arabia: A secondary analysis of the 2025 Elderly Survey
Source: Medicine (Baltimore). 2026 Oct 16;105(42):e51152. doi: 10.1097/MD.0000000000051152 (PMC13681130; doi:10.1097/MD.0000000000051152)
Supplement: Supplementary file 1 [file medi-105-e51152-s001.docx]

**Supplementary File:** Number of individuals aged ≥60 years by nationality (Saudi/Non-Saudi), and administrative region

| **Region** | **Saudi resident** | **Non-Saudi resident** | **Total** |
| --- | --- | --- | --- |
| Riyadh | 271,602 | 151,895 | 423,497 |
| Makkah | 305,392 | 210,551 | 515,943 |
| Madinah | 88,912 | 33,137 | 122,049 |
| Qassim | 61,636 | 9,978 | 71,614 |
| Eastern Province | 170,884 | 67,152 | 238,036 |
| Asir | 103,211 | 19,756 | 122,967 |
| Tabuk | 29,874 | 9,124 | 38,998 |
| Hail | 33,973 | 6,437 | 40,410 |
| Jazan | 72,079 | 19,574 | 91,653 |
| Najran | 20,441 | 6,968 | 27,409 |
| Al-Baha | 22,032 | 3,010 | 25,042 |
| Al-Jawf | 19,660 | 5,029 | 24,689 |
| Northern Borders | 13,637 | 2,793 | 16,430 |
| Total | 1,213,333 | 545,404 | 1,758,737 |
